# Conventional and emerging roles of the energy sensor Snf1/AMPK in *Saccharomyces cerevisiae*

**DOI:** 10.15698/mic2018.11.655

**Published:** 2018-09-29

**Authors:** Paola Coccetti, Raffaele Nicastro, Farida Tripodi

**Affiliations:** 1 Department of Biotechnology and Biosciences, University of Milano-Bicocca, Milan, Italy.; 2 SYSBIO, Centre of Systems Biology, Milan, Italy.; 3 Present address: Department of Biology, University of Fribourg, Fribourg, Switzerland.

**Keywords:** budding yeast, metabolism, stress response, aging, transcription, signaling, cell cycle, endocytosis, DNA damage, glucose repression

## Abstract

All proliferating cells need to match metabolism, growth and cell cycle progression with nutrient availability to guarantee cell viability in spite of a changing environment. In yeast, a signaling pathway centered on the effector kinase Snf1 is required to adapt to nutrient limitation and to utilize alternative carbon sources, such as sucrose and ethanol. Snf1 shares evolutionary conserved functions with the AMP-activated Kinase (AMPK) in higher eukaryotes which, activated by energy depletion, stimulates catabolic processes and, at the same time, inhibits anabolism. Although the yeast Snf1 is best known for its role in responding to a number of stress factors, in addition to glucose limitation, new unconventional roles of Snf1 have recently emerged, even in glucose repressing and unstressed conditions. Here, we review and integrate available data on conventional and non-conventional functions of Snf1 to better understand the complexity of cellular physiology which controls energy homeostasis.

## INTRODUCTION

Cell growth and proliferation require a high amount of energy for biosynthetic pathways. Cells take energy from nutrient intake and both unicellular and multicellular eukaryotes have evolved systems that allow dynamic sensing of energy sources, mainly sugars. The class of Snf1/AMPK (Sucrose non-fermenting/AMP-activated protein kinase) plays a key role as a guardian of cellular energy 1. They are highly conserved serine/threonine kinases and their primary role is the integration of signals regarding nutrient availability and environmental stress, ensuring the adaptation to those conditions and cell survival 2.

Here we discuss the mechanisms of action of Snf1, a member of the Snf1/AMPK family in *Saccharomyces cerevisiae* and its conventional roles in the regulation of metabolism, stress response and aging. In addition, we also focus on recent advances showing emerging functions of Snf1 on the modulation of key processes such as endocytosis and cellular trafficking as well as cell cycle, proliferation and metabolism*.*

## SNF1 COMPLEX COMPOSITION 

Protein kinase Snf1 in yeast is a heterotrimeric complex made by the catalytic α subunit Snf1, a regulatory β subunit (alternatively Gal83, Sip1 and Sip2) and the γ subunit Snf4 3.

The catalytic α subunit (encoded by the *SNF1* gene) was identified in a screening of mutants unable to grow in presence of sucrose as carbon source 4. The Snf1 subunit is constitutively expressed and constituted by a catalytic N-terminal domain and a C-terminal regulatory region. The regulatory region presents a short autoinhibitory sequence (AIS) (380 - 415 aa) and a domain which mediates the interactions with the β subunits of the complex. The autoinhibitory domain interacts with both the regulatory subunit Snf4 and the kinase domain of Snf1. The interaction with Snf4 relieves the inhibition of the AIS allowing the phosphorylation of Thr210 residue of Snf1 that determines its activation 5,6.

In *S. cerevisiae*, three β subunits (Gal83, Sip1 and Sip2) are present. They share partially redundant functions, since only the triple mutant *sip1*Δ*sip2*Δ*gal83*Δ strain shows growth defects when glycerol or ethanol are added as carbon sources 7,8. The β subunits contain a conserved C-terminal sequence in which two domains are present: the KIS domain (Kinase Interacting Sequence) that mediates the interaction with the α-subunit Snf1 9 and the ASC domain (Association with SNF1 kinase Complex) that allows the interaction with Snf4 10. Differently, the N-terminal sequence is specific for each β subunit and confers a different subcellular localization pattern to each protein. All three proteins are mainly cytoplasmic in presence of high glucose concentrations. Upon glucose depletion, Sip1 relocalizes to the vacuolar membrane, Gal83 relocalizes to the nucleus, while Sip2 remains cytoplasmic 11. Thus, the role of the β subunits is to interact with Snf1 and to modulate its subcellular localization 11,12. The particular localization of the kinase complexes with different β subunits confers specialized functions. For example, Sip1 alone is not able to sustain growth on ethanol or glycerol and determines a very low kinase activity of the complex 13, Sip2 function seems to be involved in the mechanism of cellular aging 14, while Gal83 plays its main role in the Snf1-dependent transcriptional regulation, since in low glucose it determines the nuclear localization of the Snf1 complex thank to its NLS (Nuclear Localization Signal). On the contrary, NES (Nuclear Export Signal), present on the sequence of Gal83, allows the exit from the nucleus of the complex when high glucose concentrations are available 15. In addition, Gal83 mediates the interaction of Snf1 with some substrates, such as the transcription activator Sip4 16 and the transcriptional apparatus 17. It has also been shown that deletion of the glycogen binding domain (GBD) of Gal83 leads to a constitutive activation of Snf1, which results able to modulate the expression of some Snf1-regulated genes also in high glucose concentrations 18. The GBD domain of Gal83 also interacts with the Reg1/Glc7 phosphatase complex, responsible for Snf1 inactivation 18. Taken together those data suggest that Gal83 plays a dual role regulating nuclear localization of Snf1 in low glucose and guaranteeing its inactivation in high glucose.

Similarly to *SNF1*, the gene encoding the γ subunit, *SNF4*, was identified by isolation of a sucrose non fermenting mutant 4. Snf4 is a constitutively expressed protein that binds both the α and β subunits of the Snf1 complex 10,19. The role of Snf4 is to relieve the inhibition of Snf1 interacting with its AIS domain, stabilizing the Snf1 complex in the active conformation 20. In fact, *SNF4* deletion causes a decreased kinase activity of Snf1, whereas deletion of the AIS domain of Snf1 fully complement the phenotype of a *snf4*Δ strain 19,20. Remarkably, the activating phosphorylation of Thr210 residue of Snf1 is still detectable in a *snf4*Δ strain 21 and in high glucose Snf4 seems to be required for the proper inactivation of Snf1 mediated by the phosphatase complex Reg1/Glc7 18. Thus, these findings indicate that Snf4 plays a complex role in the regulation of Snf1.

## REGULATION OF SNF1 ACTIVITY

**Figure 1:**
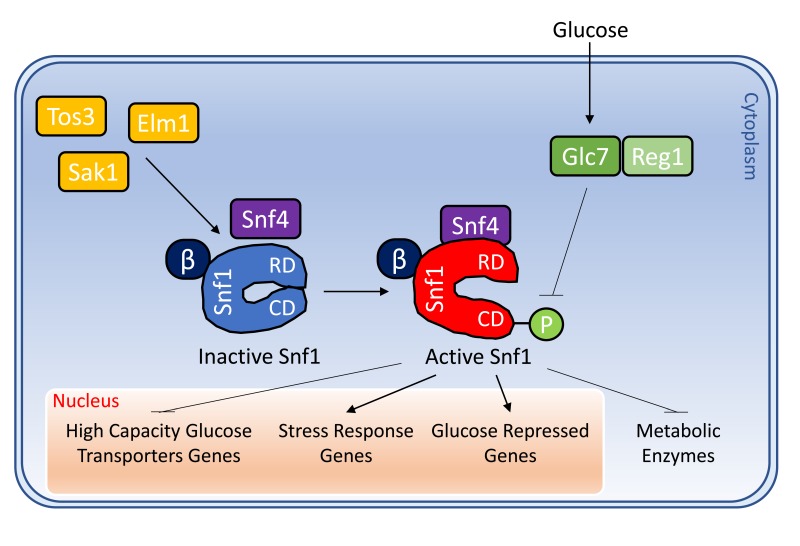
FIGURE 1: Schematization of the activation of Snf1 and its main conventional functions. Snf1 complex is composed by the α subunit Snf1, the γ subunit Snf4 and one of three alternative β subunits Gal83, Sip1 or Sip2. Snf1 is phosphorylated on T210 by the upstream kinases Sak1, Tos3 and Elm1, while it is de-phosphorylated by the phosphatase complex Glc7/Reg1. When active, Snf1 phosphorylates transcription factors which regulate the expression of genes involved in glucose transport, stress response and glucose repression. In addition, Snf1 directly phosphorylates some metabolic enzymes. See text for details.

Snf1 complex is activated through phosphorylation of the Thr210 of the α subunit by one of the three constitutively active upstream kinases Sak1, Tos3 and Elm1 22,23 (Fig. 1). This phosphorylation is essential for Snf1 activity, since the *sak1*Δ*tos3*Δ*elm1*Δ strain shows the same phenotype of a *snf1*Δ strain, such as growth defects in presence of limiting glucose or alternative carbon sources like glycerol or ethanol 22.

Although Snf1 phosphorylation is a key step for its activation, a non-phosphorylatable Snf1 mutant (Snf1-T210A) retains a low catalytic activity, originating intermediate phenotypes 24,25. Also, the mutation of the lysine which constitutes the ATP binding site in the kinase domain (Snf1-K84R), which for many aspects mimics the loss of Snf1 protein, still confers a slight catalytic activity 26,27,28,29.

On the other side, in response to high glucose concentrations Snf1 is inactivated through dephosphorylation of Thr210 by the Gcl7 protein phosphatase (also known as PP1), which is targeted to Snf1 by the adaptor subunit Reg1 30,31. Reg1 interacts both with Glc7 and Snf1 when glucose is largely available in the culture medium and loss of Reg1 leads to the constitutive activation of Snf1 32,33,34. It has been reported that in high glucose concentration, Hxk2 (Hexokinase 2) regulates the activity of PP1 and consequently the activation of Snf1 kinase 31.

Active Snf1 phosphorylates serine and threonine residues contained in the consensus pattern Φ-x-R-x-x-S/T-x-x-x-Φ, where Φ is a hydrophobic residue 35.

Differently from its mammalian homolog AMPK, yeast protein kinase Snf1 is not allosterically activated by AMP 36. However, it was demonstrated that ADP molecules are able to bind the γ subunit Snf4, preventing Snf1 dephosphorylation mediated by Glc7 37,38.

The structure of the kinase domain of Snf1 showed that it is a dimer, which represents an inactive form of the kinase, since Thr210 is inaccessible for phosphorylation by the activating kinases 39. Although these results suggest the existence of another layer of regulation of Snf1 activity, further investigation is required to better elucidate its physiological relevance.

Interestingly, some recent evidence indicates additional mechanisms that regulate Snf1 activity: *(i)* phosphorylation of Ser214, inside the activation loop, downregulates Snf1 function 40; *(ii)* SUMOylation of the catalytic α subunit Snf1 inhibits its activity, possibly by attenuating its levels in the cell and/or favoring the inactive conformation of the kinase 41; *(iii)* the SAGA acetyl transferase complex deubiquitylates Snf1 affecting the stability of the complex and its kinase activity 42;* (iv)* the ubiquitin-associated motif (UBA) of the α subunit Snf1 indirectly regulates *SNF1* gene expression and Snf1 interaction with the γ subunit Snf4 43.

## NETWORK OF SNF1 PHYSICAL INTERACTORS

**Figure 2:**
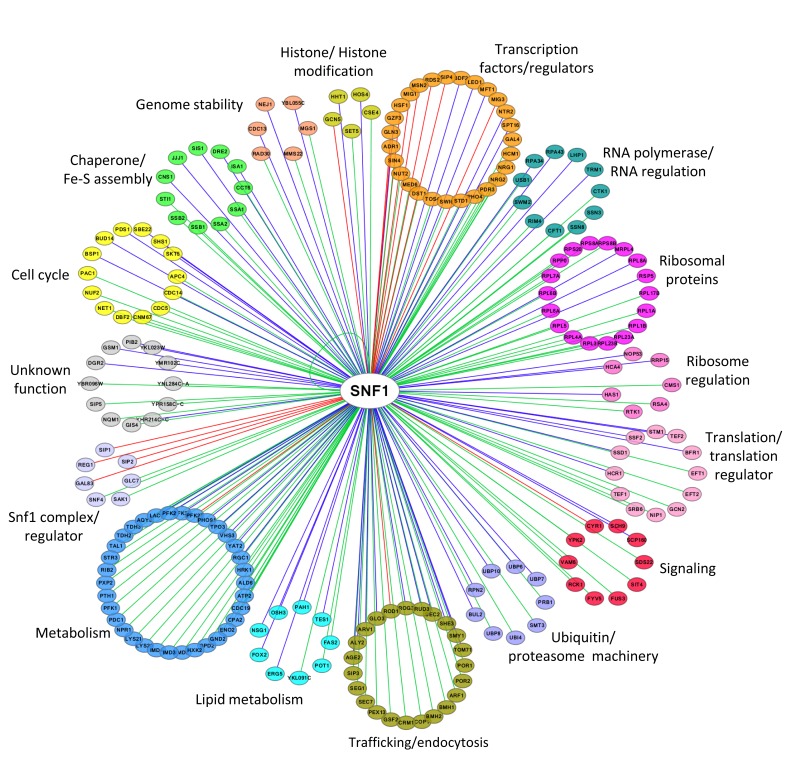
FIGURE 2: Network of Snf1 physical interactors. The network reports the known physical associations obtained from SGD (Saccharomyces Genome Database, http://www.yeastgenome.org. Interactors are clustered according to their function and colored differently. When the interactor is also a substrate of Snf1 according to the Yeast Kinase Interaction Database (KID, http://www.moseslab.csb.utoronto.ca/KID; 44), the edge is colored in red if phosphorylation was analyzed by low throughput assays (LTP *in vitro* kinase assays; *in vitro* phosphorylation site mapping; *in vivo* phosphorylation site mapping; phosphorylation reduced or absent in kinase mutant) or in blue if phosphorylation was assayed only by high throughput analysis (protein chip data for *in vitro* phosphorylated substrate; HTP *in vitro* phosphorylation). Data visualization and analysis was performed with Cytoscape 45.

A total of 216 proteins physically interacting with Snf1 are annotated in SGD (Saccharomyces Genome Database, http://www.yeastgenome.org, 92 of which are also Snf1 substrates (identified by high throughput or low throughput assays). In Figure 2 we clustered them on the base of their function. Apart from the most known interactors involved in Snf1 complex regulation, transcription, histone modification, signaling and metabolism, there are many proteins which regulate translation, ribosome function, intracellular transport/trafficking and cell cycle. In addition, some of them are also proteins of the ubiquitin/proteasome machinery, chaperones and Fe/S cluster proteins (Fig. 2). Remarkably, only a few of them have been extensively investigated for the physiological relevance of Snf1-dependent phosphorylations, suggesting that many functions of Snf1 are still to be discovered.

## CONVENTIONAL ROLES OF SNF1

### Snf1 and the regulation of transcription

The most studied function of Snf1 is the regulation of transcription, involving more than 400 genes 46. Snf1 acts both on transcription factors and on chromatin remodeling 47,48,49,50, as also highlighted by the number of its interactors belonging to these two classes of proteins (Fig. 2).

Mig1 is the most important glucose-regulated transcriptional repressor 51. Mig1 is phosphorylated by Snf1 on four sites when glucose is scarce, causing the activation of a NES (Nuclear Export Signal) sequence that causes its translocation from the nucleus to the cytoplasm through the exportin Msn5 48,52,53. Important in the regulation of Mig1 is hexokinase Hxk2, which interacts with the transcriptional repressor directly in the nucleus to avoid its phosphorylation by Snf1, thus providing a link between glucose metabolism and transcription of glucose-repressed genes 54. Mig1 represses about 90 genes, including those coding for enzymes required for the metabolism of sucrose (*SUC2*), maltose (the *MAL* regulon) and galactose (*GAL4*) 55. Furthermore, Mig1 controls the expression of high-affinity glucose transporters, required when glucose is scarce (*HXT2*, *HXT4*) 56, represses *TPS1*, essential for the metabolism of trehalose 57 and genes coding for enzymes of the TCA cycle 58.

Besides Mig1, Snf1 regulates the activity of other transcription factors. Cat8 and Sip4, which bind Carbon Source Responsive Elements (CSRE), regulate the expression of gluconeogenic genes 59 and are activated by Snf1 phosphorylation 49,60. Cat8 activates the expression of glucose-repressed genes alongside transcription factor Adr1, which is itself a target of Snf1 61,62. Moreover, in a fine mechanism of positive feedback, the *CAT8* gene is activated by Snf1 through inhibition of Mig1 47. In addition, Gcn4, the transcription factor responsible for the expression of genes involved in amino acid biosynthesis, is also regulated by Snf1 when in complex with the β subunit Gal83 or Sip1, but not Sip2 63.

Snf1 has been reported to phosphorylate Ser10 of histone H3 and to promote the acetylation on Lys14 of histone H3 by Gcn5, a component of the SAGA complex 50. Snf1-mediated regulation of histone H3 is involved in the expression of *ADY2 *gene. In fact, Snf1 stimulates the binding of Gcn5 and the acetylation of histone H3 at *ADY2* promoter, promoting the transcription of this gene 64.

### Snf1 and the regulation of metabolism

Besides its role in regulating the transcription of several genes involved in metabolism, Snf1 directly regulates, through phosphorylation, important metabolic enzymes. In fact, together with the class of transcription factors and regulators, proteins linked to metabolism are the most abundant among Snf1 interactors (Fig. 2). Probably the most impactful function exerted by Snf1 as a direct regulator of metabolism is the regulation of the acetyl-CoA carboxylase Acc1 65. In yeast, loss of Snf1 causes a dramatic accumulation of fatty acids and the carbon overflow into the fatty acid biosynthetic pathway has been shown to cause inositol auxotrophy mediated by the impairment of *INO1* expression 65,66. Moreover, the excessive allocation of carbon into fatty acids causes a depletion of the intracellular acetyl-CoA pool, and thus a global reduction of acetylation of histones, of Swi4, the DNA-binding protein of the transcription factor SBF 67, and of the β-subunit Sip2 68.

Snf1 was also shown to phosphorylate Pfk27, the second isoform of 6-phosphofructo-2-kinase 69. Upon glucose removal, Snf1 phosphorylates Pfk27 in its N-terminal domain, leading to the SCF^Grr1^-dependent degradation of Pfk27 69. In particular, Snf1-dependent phosphorylation is required to promote Pfk27 association with the F-box protein Grr1 69, thus leading to Pfk27 turnover and consequently to a reduction of fructose-2,6-bisphosphate. The importance of Pfk27 turnover is highlighted by the fact that expression of a non-phosphorylatable and non-degradable Pfk27 protein inhibits growth on glycerol 69.

Moreover, Snf1 phosphorylates Gpd2, the glycerol-3-phosphate dehydrogenase required for anaerobic growth, thus inhibiting glycerol synthesis during the diauxic shift. In fact, it was reported that Snf1 phosphorylates Gpd2 on Ser72 priming Gpd2 for subsequent phosphorylation on Ser75, probably by Yck1 70.

### Snf1 and PKA crosstalk

In yeast, the main pathway activated by glucose is the PKA pathway, involved in metabolism, growth and proliferation 71,72,73. Targets of PKA include glycolytic and gluconeogenetic enzymes, proteins involved in the metabolism of storage carbohydrates, transcription factors regulating stress response, ribosomal biogenesis, and carbohydrate metabolism. Active PKA directly stimulates glycolysis, cell growth and cell cycle progression, at the same time gluconeogenesis, stress resistance and mobilization of glycogen and trehalose are down-regulated 71,74. Several examples of cross-talk between Snf1 and PKA pathways have been reported 75. Indeed, both kinases regulate the activity of the same transcription factors. Adr1, the transcriptional activator of glucose-repressed genes, is inactivated by PKA and activated by Snf1 which promotes its phosphorylation 76,77. Msn2, the stress-responsive transcriptional activator, which is a well-known target of PKA, is phosphorylated also by Snf1 in glucose starvation 78. PKA indirectly controls the localization of the β subunit Sip1 and, as a consequence, of the Snf1-Sip1 complex 12. In addition, PKA contributes to regulate Sak1, one of the Snf1-activating kinases 79.

Notably, recent data nicely complement observations of a cross-talk between Snf1 and PKA. Indeed, the adenylate cyclase Cyr1 and Snf1 interact in a nutrient-independent manner 29. Active Snf1 phosphorylates Cyr1 and negatively regulates cAMP content and PKA-dependent transcription 29. Moreover, loss of Snf1 causes an alteration in the phosphorylation pattern of adenylate cyclase 29, suggesting that the crosstalk between Snf1 and PKA is more complex than actually reported and needs to be further investigated.

### Snf1 and the regulation of TORC1

The Target Of Rapamycin Complex I (TORC1) is a highly conserved nutrient-responsive regulator of cell growth and metabolism in all eukaryotes 80,81,82. Contrary to AMPK, which is active under nutrient-poor conditions, TORC1 is active under nutrient-rich conditions in budding yeast and also in the presence of growth factors in higher eukaryotes 83,84. In yeast, TORC1 is composed of Tor1, Kog1/Raptor, Lst8 and Tco89 81,85,86. Kog1/Raptor is known to recruit substrates such as 4EBP1 and ribosomal S6 kinase (S6K) to the TORC1 complex 87,88 and is required for the regulation of its activity 80,89.

Kog1 is phosphorylated by Snf1 under glucose deprivation, as AMPK does on the ortholog Raptor 90, confirming a conserved regulatory function of Snf1/AMPK on TORC1 complex. Nevertheless, the role of Snf1-dependent phosphorylation on Kog1 is somehow different, since Snf1 phosphorylation stimulates the dissociation of the Kog1-Tor1 complex and the formation of Kog1-bodies by limiting the level of active TORC1 complex in the cell 90. Thus, although the final result of Snf1 phosphorylation is the inactivation of TORC1 activity, this is reached by increasing the activation threshold of TORC1 to guarantee a cellular commitment to a quiescent state and then survival in starvation condition 90.

In the presence of nutrients, TORC1 phosphorylates and activates Sch9, the ortholog of S6K in yeast which, together with other substrates, drives ribosome biosynthesis 91,92,93. Through an *in vitro* kinase assay and epistasis analysis, Sch9 has been shown to be also a target of Snf1, indeed total Sch9 phosphorylation is reduced in *snf1*Δ mutant 94. On the other hand, Snf1-hyperactive cells display a dramatic decrease of TORC1 activity 95. Moreover, Snf1 activity is required for the downregulation of TORC1-dependent phosphorylation on Sch9 also in glucose deprivation 96.

Interestingly, Orlova and coworkers showed that rapamycin treatment results in a significant increase of Thr210 phosphorylation on Snf1 97, suggesting a reciprocal regulation of Snf1 by TORC1 and a more complex crosstalk between the two signaling pathways.

TORC1 activity is involved in the regulation of autophagy, a cellular recycling system that degrades proteins and organelles by delivery to the vacuole in response to nutrient deprivation 98,99. In yeast, nitrogen starvation, which induces TORC1 inhibition, Atg13 dephosphorylation as well as Atg1 phosphorylation, results in activation of autophagy 100. Remarkably, although Snf1 has been proposed as a positive regulator of nitrogen-induced autophagy probably because of its phosphorylation on Atg1 101, in *snf1*Δ cells, the translocation of GFP-Atg8 to the vacuole is reduced by 50% compared to the wild type 102. Moreover, Snf1 activity is essential for glucose starvation-induced autophagy, and mitochondrial respiration is a required feature for this energy deprivation condition 102. These interesting results indicate that further investigations are required to better elucidate the different mechanisms which regulate nitrogen- and glucose-induced autophagy, as well as how Snf1 is involved in such a regulation.

### Snf1 and the regulation of stress response

Besides nutritional deprivation, Snf1 is also involved in the response to other cellular stresses. Snf1 activity protects against toxicity caused by cadmium 103, hygromycin B 26, hydroxyurea 24, selenite 104, and iron 105. Snf1 also regulates HSF (Heat Shock transcription Factor) ensuring the cellular resistance to high temperature, oxidative stress 106,107 and counteracts the activity of the transcriptional inhibitor Nrg1, promoting the expression of *ENA1*, responsible for Na^+^ ions detoxification 108.

In addition, protein kinase Snf1 regulates the Unfolded Protein Response (UPR), the evolutionary conserved pathway activated when improperly folded proteins accumulate and induce endoplasmic reticulum (ER) stress 109,110. ER misfunction causes severe disease conditions 110, thus the elucidation of the molecular mechanism by which AMPK regulates UPR signaling attracts the increasing interest of cell biologists. Nevertheless, the role of Snf1 in this pathway is still not clear, since partially discrepant data were published on yeast 111,112. Mizumo and coworkers support a negative role of Snf1 in such a regulation, showing that the deletion of *SNF1 *gene and Snf1 activation cause increased and decreased resistance to ER stress, respectively 112. On the contrary, although results from Casamayor’s laboratory highlight that Snf1 activation induces hypersensitivity to ER-stress-inducer agents 111, they also reported that *snf1*Δ cells are more sensitive to tunicamycin, a known inducer of ER stress 113.

Thus, even though these data indicate an interesting role for Snf1 in the regulation of ER stress response, more work is needed to understand the underlying molecular mechanism.

### Snf1 and the regulation of DNA damage

Interesting results from Simpson-Lavy and collaborators recently show cross-talk between Snf1 and protein kinases involved in DNA damage (Mec1/ATR and Tel1/ATM) 114. Phosphorylation of the SUMO E3 ligase Mms21 by Mec1 and Tel1 induces SUMOylation and inactivation of Snf1, in response to DNA damage. Thus, fermentation increases while respiration is switched off. Remarkably, inactivation of Snf1 activity by SUMOylation does not affects its phosphorylation at Thr210, indicating that SUMOylation and phosphorylation of Snf1 are independently regulated. The authors suggest that this metabolic switch may protect yeast cells from oxidative stress and propose interesting parallelisms with Warburg effect in cancer cells 114.

### Snf1 and the regulation of aging

Given the role of AMPK kinase in the regulation of energy homeostasis, it is not surprising the existence of a strong relationship between aging and the AMPK pathway 115,116,117,118,119. Indeed, hallmarks of the aging process such as mitochondrial dysfunction 120, autophagy 121, endoplasmic reticulum stress 122 and DNA damage repair 123 are regulated by AMPK. Importantly, both pharmacological stimulation and exercise increase AMPK activity in skeletal muscle of young rats but not in old ones 124. Moreover, AMPK-dependent acetyl-CoA carboxylase and mitochondrial biogenesis are impaired with aging 124. These and other results suggest that the basal activity of AMPK declines with aging, contributing to the dysregulation of intracellular metabolism.

The first evidence of the involvement of Snf1/AMPK in the aging process in yeast occurred several years ago when Asharafhi and co-workers discovered that Snf1 activity increased in replicative aging even in the presence of abundant glucose in the environment 14. Moreover, loss of the β-subunit Sip2 accelerates aging 14 and Sip2 acetylation enhances its interaction with Snf1, decreases the kinase activity of the complex and extends Replicative Life Span (RLS, an aging model of mitotically active cells) 94. Thus, in a yeast model of replicative aging, these results indicate that Snf1 inactivation promotes longevity 94.

On the contrary, in a yeast model of Chronological Life Span (CLS, a model of aging in post-mitotic mammalian cells), Snf1 activity is critical for the extension of CLS in caloric restriction condition and cells deleted for *SNF1* gene have a very short CLS 125,126. Accordingly, the upregulation of Snf1 activity extends the life span 127 and drugs like metformin (which activates AMPK) are being proposed for the treatment of age-associated disorders 128.

Taken together these data indicate that Snf1 activity promotes longevity in CLS while accelerates RLS, showing distinct and opposite mechanisms in the regulation of aging, as also reported for other proteins in yeast 129,130.

## NON CONVENTIONAL ROLES OF SNF1

### Snf1 and the regulation of endocytosis

Recent data suggest that Snf1 function is not limited to conditions of carbon limitation. One of such functions is the regulation of proteins involved in endocytosis and cellular trafficking, indeed several interactors and substrates of Snf1 are in this cluster (Fig. 2). Snf1 interacts with and phosphorylates the α-arrestin Rod1 27,131,132, which regulates endocytosis of the lactate transporter Jen1 and of the hexose transporters Hxt1, Hxt3 and Hxt6, in response to glucose. Remarkably, Snf1 phosphorylation on Rod1 occurs not only in glucose limitation, but also in the presence of glucose. In this condition, Snf1 phosphorylation inhibits Rod1-mediated trafficking of Hxt1 and Hxt3, thus maintaining a high glucose transporter activity 133. Therefore, Snf1-mediated phosphorylation has both an inhibitory and activatory function on the trafficking of hexose transporters, depending on the level of Snf1-mediated phosphorylation on Rod1 133. According to the proposed model, when Snf1 activity is high, Rod1 is hyper-phosphorylated and the endocytosis of hexose transporters is active. On the other hand, in glucose growing cells, Snf1 activity is low, Rod1 is hypo-phosphorylated and thus its trafficking function is inhibited, indicating that Snf1 retains physiologically important function also under high-glucose conditions, probably to direct its activity to specific targets 133.

Snf1 also regulates Arf3 134, one of the ADP-ribosylation factors (Arfs), involved in vesicle transport and actin reorganization. Yeast Arf3 is required for invasive growth and its activity is stimulated upon glucose-depletion in a Snf1-dependent manner. The regulation of invasive growth in nutrient depletion is actually a conventional role of Snf1 135,136. However, the peculiarity of the activation of Arf3 is that it does not depend on Snf1 kinase activity, but rather on its absolutely new role as a GEF (Guanine Nucleotide Exchange Factor). In fact, it was shown that the C-terminal hydrophobic α-helix core of Snf1 is a non-canonical GEF for Arf3 activation 134.

### Snf1 and the regulation of cell proliferation, cell cycle and metabolism

Several reports indicate that Snf1 could be active in the presence of high glucose 54,133,137,138, indicating regulatory roles for Snf1 under glucose repression. In facts, cells lacking Snf1 (both *snf1*Δ and *snf1as* mutant, whose activity can be chemically inhibited) show a slow growth phenotype and an increased fraction of cells in G1 phase, in synthetic medium supplemented with 2% glucose 25,139. Nevertheless, no perturbations of growth occur in complete (YPD, with 2% glucose) and in synthetic media with 5% glucose 25,139, as well as in complete synthetic medium (our unpublished results), indicating that the nutritional composition of the media may influence the cellular requirement for Snf1 function.

**Figure 3:**
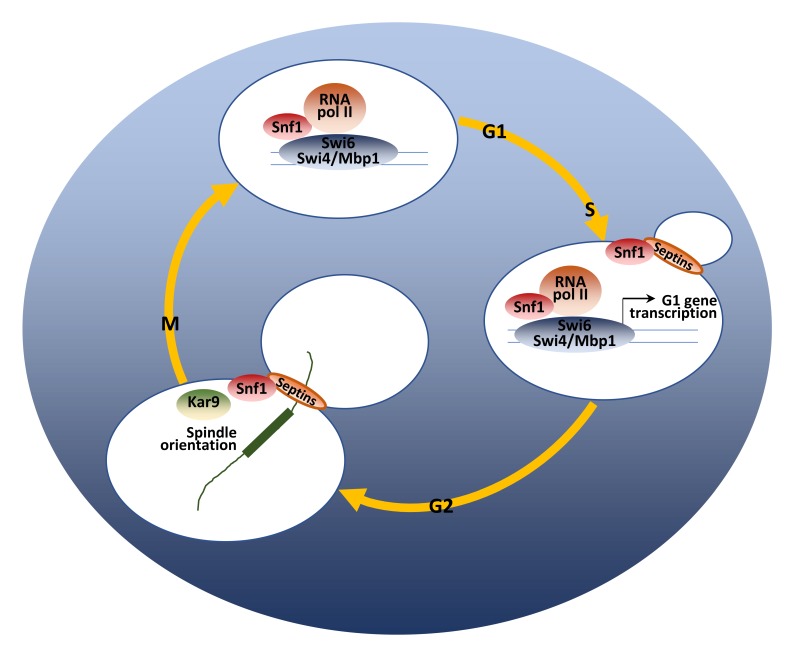
FIGURE 3: A model of the regulatory role of Snf1 during the cell cycle. At the G1/S phase transition, Snf1 promotes the binding of Swi4, Mbp1 and Swi6 proteins to G1 promoters and favors the proper recruitment of the RNA Polymerase II. From bud emergence, active Snf1 is localized to the bud neck, in a septin-dependent manner. At the metaphase-to-anaphase transition, Snf1, as part of the Kar9-dependent pathway, promotes spindle alignment along the mother-bud axis and guarantees proper nuclei segregation during mitosis. See text for details.

The relevant role of the catalytic activity of Snf1 in regulating proliferation in glucose-repressed condition is further supported by the fact that Snf1 loss reduces the expression of G1-specific genes 139. The G1/S transition is regulated by the expression of about 150 genes (the G1 regulon) 140, controlled by the transcription factors SBF (Swi4-Swi6) and MBF (Mbp1-Swi6) 67. Snf1 directs the expression of both SBF and MBF-regulated genes 25,139, by modulating the recruitment of both the co-activator Swi6 and the RNA polymerase II to the promoters of G1-genes 139 (Fig. 3).

Snf1-T210 is weakly phosphorylated in 2% glucose, confirming that it is partially active in that growth condition 21,139,141. Consistently, the non phosphorylatable *SNF1-T210A* mutant shows a slow growth phenotype and a delayed G1/S transition. 25,139.

Snf1 exerts its function in mitosis too and active Snf1 is localized at the division site from the time of bud emergence to cytokinesis 142. Both septins and protein kinase Elm1 are required for proper Snf1 localization to the bud neck, indicating that the presence of an accurate scaffold is necessary for this process (Fig. 3). Loss of Snf1 activity causes a defect in the correct alignment of the mitotic spindle, that in turn induces a delay of the metaphase-to-anaphase transition, thus clearly indicating Snf1 function for proper spindle orientation. Two major pathways are responsible for the spindle alignment along the mother-bud axis in budding yeast: the Kar9-pathway and the Dyn1-pathway 143. Recent results show that Snf1 acts in parallel to Dyn1 and in concert with Kar9 to promote spindle positioning, probably phosphorylating components of the Kar9-dependent pathway 142. In support of these data, several key regulators of cell cycle are known interactors of Snf1, mainly involved in the network of spindle orientation, mitotic exit and cytokinesis (Fig. 2).

It is amazing that cells lacking Snf1 and growing in synthetic media containing 2% glucose, show an extensive transcriptional reprogramming, being the most upregulated genes mainly involved in transmembrane transport and metabolic processes such as aminoacid biosynthesis, iron homeostasis and redox metabolism 144. Moreover, an increase of cellular dependence on mitochondrial function in glucose repression condition is clearly noticeable in *snf1*Δ cells, further supporting the emerging roles of Snf1 in non-limiting nutrient condition too 144.

## PERSPECTIVES AND CONCLUDING REMARKS 

Many studies have reported that AMPK activity is altered in several diseases, such as inflammation, diabetes and cancer 145,146. Moreover, the number of pharmacological agents that activate AMPK has continued to increase and some of them are promise hypoglycemic agents. Importantly, although AMPK is considered a key target for cancer treatment, emerging data indicate that AMPK performs both anti- and pro-tumorigenic roles depending on the composition of AMPK complex, signaling networks and environmental conditions 147,148. The pro-tumorigenic role of AMPK involves promotion of metabolic adaptation for cancer cell survival by regulating fatty acid metabolism and maintaining the ability to growth in stressful conditions 145,149.

Therefore, the expansion of the repertoire of AMPK substrates, as well as more in-depth studies of the molecular mechanisms by which AMPK is activated, will help to better understand the roles of this kinase in the regulation of human pathologies. In this context, the yeast unicellular organism *Saccharomyces cerevisiae* is a powerful model for studying fundamental aspects of eukaryotic cell biology and to validate the increasing downstream targets of the class of Snf1/AMPK protein kinases which control the complexity of cellular physiology.
